# Long-term and large-scale multispecies dataset tracking population changes of common European breeding birds

**DOI:** 10.1038/s41597-021-00804-2

**Published:** 2021-03-26

**Authors:** Vojtěch Brlík, Eva Šilarová, Jana Škorpilová, Hany Alonso, Marc Anton, Ainars Aunins, Zoltán Benkö, Gilles Biver, Malte Busch, Tomasz Chodkiewicz, Przemysław Chylarecki, Dick Coombes, Elisabetta de Carli, Juan C. del Moral, Antoine Derouaux, Virginia Escandell, Daniel P. Eskildsen, Benoît Fontaine, Ruud P. B. Foppen, Anna Gamero, Richard D. Gregory, Sarah Harris, Sergi Herrando, Iordan Hristov, Magne Husby, Christina Ieronymidou, Frédéric Jiquet, John A. Kålås, Johannes Kamp, Primož Kmecl, Petras Kurlavičius, Aleksi Lehikoinen, Lesley Lewis, Åke Lindström, Aris Manolopoulos, David Martí, Dario Massimino, Charlotte Moshøj, Renno Nellis, David Noble, Alain Paquet, Jean-Yves Paquet, Danae Portolou, Iván Ramírez, Cindy Redel, Jiří Reif, Jozef Ridzoň, Hans Schmid, Benjamin Seaman, Laura Silva, Leo Soldaat, Svetoslav Spasov, Anna Staneva, Tibor Szép, Guido Tellini Florenzano, Norbert Teufelbauer, Sven Trautmann, Tom van der Meij, Arco van Strien, Chris van Turnhout, Glenn Vermeersch, Zdeněk Vermouzek, Thomas Vikstrøm, Petr Voříšek, Anne Weiserbs, Alena Klvaňová

**Affiliations:** 1 Czech Society for Ornithology, Prague, Czechia; 2 Department of Ecology, Faculty of Science, Charles University, Prague, Czechia; 3 Czech Academy of Sciences, Institute of Vertebrate Biology, Brno, Czechia; 4 Portuguese Society for the Study of Birds (SPEA), Lisbon, Portugal; 5 Catalan Ornithological Institute, Natural History Museum of Barcelona, Barcelona, Spain; 6 Latvian Ornithological Society, Riga, Latvia; 7 Department of Zoology and Animal Ecology, Faculty of Biology, University of Latvia, Riga, Latvia; 8 Romanian Ornithological Society, Cluj-Napoca, Romania; 9 Evolutionary Ecology Group, Hungarian Department of Biology and Ecology, Babeş-Bolyai University, Cluj-Napoca, Romania; 10 Ministère de l’Environnement, du Climat et du Développement durable, Luxembourg, Luxembourg; 11 Dachverband Deutscher Avifaunisten (DDA), Muenster, Germany; 12 Museum & Institute of Zoology, Polish Academy of Sciences, Warszawa, Poland; 13 Polish Society for the Protection of Birds (OTOP), Marki, Poland; 14 BirdWatch Ireland, on behalf of the National Parks & Wildlife Service, Kilcoole, Republic of Ireland; 15 MITO2000, Parma, Italy; 16 SEO/BirdLife, Madrid, Spain; 17 Aves-Natagora, Namur, Belgium; 18 DOF/BirdLife Denmark, Copenhagen, Denmark; 19 UMR7204 CESCO, MNHN-CNRS-SU, Paris, France; 20 Sovon Dutch Centre for Field Ornithology, Nijmegen, The Netherlands; 21 Department of Animal Ecology & Ecophysiology, Institute for Water and Wetland Research, Radboud University, Nijmegen, The Netherlands; 22 RSPB Centre for Conservation Science, Sandy, United Kingdom; 23 Department of Genetics, Evolution and Environment, Centre for Biodiversity & Environment Research, University College London, London, United Kingdom; 24 British Trust for Ornithology, Thetford, United Kingdom; 25 Bulgarian Society for the Protection of Birds/BirdLife Bulgaria, Sofia, Bulgaria; 26 Section of Science, Nord University, Levanger, Norway; 27 BirdLife Norway, Trondheim, Norway; 28 BirdLife Cyprus, Nicosia, Cyprus; 29 Norwegian Institute for Nature Research, Trondheim, Norway; 30 Department of Conservation Biology, University of Göttingen, Göttingen, Germany; 31 DOPPS BirdLife Slovenia, Ljubljana, Slovenia; 32 Lithuanian Ornithological Society, Vilnius, Lithuania; 33 Vytautas Magnus University, Kaunas, Lithuania; 34 The Finnish Museum of Natural History, University of Helsinki, Helsinki, Finland; 35 Department of Biology, Lund University, Lund, Sweden; 36 Hellenic Ornithological Society, Athens, Greece; 37 Birdlife Estonia/Estonian Ornithological Society, Tartu, Estonia; 38 BirdLife International, Cambridge, United Kingdom; 39 Centrale ornithologique, natur&ëmwelt a.s.b.l., Kockelscheuer, Luxembourg; 40 Institute for Environmental Studies, Faculty of Science, Charles University, Prague, Czechia; 41 Department of Zoology, Faculty of Science, Palacky University, Olomouc, Czechia; 42 Slovak Ornithological Society/BirdLife Slovakia, Bratislava, Slovak Republic; 43 Swiss Ornithological Institute, Sempach, Switzerland; 44 BirdLife Austria, Vienna, Austria; 45 LIPU – BirdLife Italia, Parma, Italy; 46 Statistics Netherlands, The Hague, The Netherlands; 47 Institute of Environmental Sciences, University of Nyíregyháza, Nyíregyháza, Hungary; 48 MME/BirdLife, Budapest, Hungary; 49 INBO, Brussels, Belgium

**Keywords:** Biodiversity, Population dynamics

## Abstract

Around fifteen thousand fieldworkers annually count breeding birds using standardized protocols in 28 European countries. The observations are collected by using country-specific and standardized protocols, validated, summarized and finally used for the production of continent-wide annual and long-term indices of population size changes of 170 species. Here, we present the database and provide a detailed summary of the methodology used for fieldwork and calculation of the relative population size change estimates. We also provide a brief overview of how the data are used in research, conservation and policy. We believe this unique database, based on decades of bird monitoring alongside the comprehensive summary of its methodology, will facilitate and encourage further use of the Pan-European Common Bird Monitoring Scheme results.

## Abstract (summary)

**Table:** 

Measurement(s)	population
Technology Type(s)	Count
Factor Type(s)	time scale • geographic location
Sample Characteristic - Organism	Aves
Sample Characteristic - Location	Europe

Machine-accessible metadata file describing the reported data: 10.6084/m9.figshare.13240760

## Background & Summary

Biodiversity declines have multiple negative implications for entire ecosystems. Trophic interactions between organisms may be altered, functional diversity reduced and potential vulnerability to biodiversity loss increased due to these negative changes^1^. Despite the importance of biodiversity for human well-being^2^ and awareness of this fact by international authorities (https://www.cbd.int/sp/targets/), a recent study shows an ongoing loss of biodiversity in the last few decades^3^. Therefore, evaluation of previous conservation efforts as well as the production of robust datasets for future assessments is essential to better understand and reverse these negative trends, and to understand positive trends at the same time.

Datasets that cover long term population changes across species distribution ranges are crucial to capture variability in processes behind recent biodiversity losses and to enable precise targeting of any necessary remedial conservation actions. However, collecting long-term and broad-scale information on population changes for multiple species is challenging for various reasons. In particular, there are limited financial resources to support such efforts and limited human resources with adequate skills and training in the identification of species and systematic data collection^4^.

**Fig. 1:**
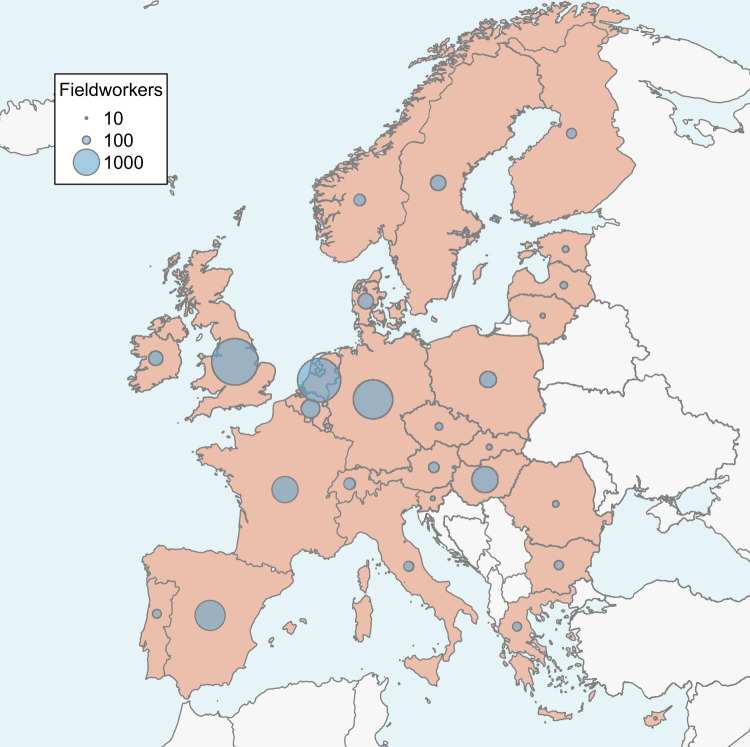
Countries providing the national bird monitoring scheme(s) outputs to the Pan-European Common Bird Monitoring Scheme (orange) and the number of fieldworkers in the national monitoring schemes (dot size; dots in the legend represent the respective number of collaborators).

Broad interest in birds worldwide has the potential to overcome some of these challenges^5^. Amateur birdwatchers are often well-trained in bird identification and many of them are willing to participate in citizen science projects aiming to better understand diverse natural processes^4,6,7^. In recent decades, numerous projects acquiring long-term and broad-scale population data on the relative abundance of birds have been established, especially in many European countries^8^ and North America^9^. These networks of fieldworkers annually collect data on species’ presence and abundance, in various habitats across numerous study plots, used for a wide range of applications^10^ (Fig. 1).

The Pan-European Common Bird Monitoring Scheme (PECBMS)^11^ uses data from the national bird monitoring schemes in Europe annually to produce a continental dataset on relative changes in breeding bird population sizes. So far, PECBMS has produced 15 annual updates of the between-year and long-term changes of widespread and common bird species breeding in Europe^10,12–14^. These data have been used for the evaluation of conservation efforts^15^, investigation of various research questions^12,16–27^ and for reporting on international biodiversity targets^28,29^. In addition, the PECBMS dataset is regularly used for assessments like the Living Planet Index (https://livingplanetindex.org/), helping to shed light on the global biodiversity state of vertebrates;^30,31^ for policy purposes by the European Environmental Agency, European Commission, Eurostat (EU headline indicators)^32^; and by Forest Europe and the Organisation for Economic Co-operation and Development to report against national and international environmental and biodiversity frameworks and goals.

Here, we present the database of the between-year and long-term relative population size changes in 170 bird species collected by thousands of volunteers in 28 European countries over the past decades^8^. The publicly available datasets can be used for numerous goals and especially for investigating research questions, conservation efficiency assessments and for policy purposes.

## Methods

### National breeding bird monitoring schemes

Fieldworkers record all, or a fixed, pre-defined set, of bird species heard or seen in the main breeding season in 28 European countries on an annual basis (Fig. 1). All observations are recorded following a scheme-specific standardized protocol based on established field methods for counting birds: point count transect, line transect, or territory or spot mapping^10,33,34^. Here, we provide a short description of the field methods used, as each scheme provides its fieldworkers with specific fieldwork instructions and training.

1. Point counts: A fieldworker counts all detected birds at census points, often placed along a transect (typically >200 meters apart) during a fixed time period to sample birds in a defined study area. Each point is usually visited twice a year.
2. Line transects: A fieldworker moves along a transect and records all detected birds along the predefined path to sample birds in a defined study area. Each transect is usually visited twice a year.
3. Territory or spot mapping: A fieldworker records all birds showing territorial behaviour in a defined study area and marks their positions and their territorial behaviour on a map. The study area is visited multiple times a year (usually 5–12) to map breeding bird territories based on the individual species-specific behaviour recorded. The species counts reflect the number of present territories.

National scheme coordinators provide all fieldworkers with instructions with the prescribed number and timing of survey visits, and information on how to record observations in terms of sampling effort, time of day, seasonality and weather conditions. This ensures the temporal and spatial consistency of data quality within individual national schemes^35^. The standardization of conditions during counting then enables unbiased comparison of results between years and individual study sites within each country.

For the selection of sampling plots, national monitoring schemes use either random, stratified random, systematic selection, or allow a free choice by fieldworkers^8,34^. Sampling plots are selected randomly within the study boundaries using a random selection method or randomly within the stratum under the stratified random method. Under these methods, study plot selection is conducted by random generators (by computer programs) and stratum is predefined as a region with similar attributes; these might be proportions of habitat types, altitude bands, bird abundance, accessibility of survey sites, or fieldworker density, depending on the local circumstances. Systematic selection predefines a spatial grid for sampling plot selection while free choice enables fieldworkers to select their study areas without restrictions^34^. The use of a free choice, or stratified random selection of sampling plots may result in a biased sampling of specific habitat types (typically species-rich habitats) and regions (remote areas poorly covered), but post-hoc stratification and weighting procedures are generally used to correct for unequal sampling and reduce sampling bias as long as the number of plots per stratum is sufficient^36^. Moreover, national coordinators provide fieldworkers with recommendations or oversee the study plot selection to prevent oversampling of specific habitat types and regions. Detailed information on scheme-specific counting protocols, study plot selection and breeding period specification can be found for each national monitoring scheme^8^.

### National species indices

A species annual index reflects population size change relative to the population size in the reference year. On an annual basis, coordinators of the national monitoring schemes produce species indices for recorded species using a tailor-made implementation of loglinear regression models (TRIM models – Trends and Indices for Monitoring data) from time series of recorded species counts at the study plots^37,38^. Species counts from a study plot reflect mean (or maximum) of individuals recorded during visits at the study plot when using point counts or line transects. For some species, only the number of individuals recorded on the second visit is used because the period of the first visit coincides with the migratory period and consequently the mean number of recorded individuals might not reflect the number of breeding individuals. The method to estimate the species counts in a plot is constant within a national scheme.

Missing data occur in the species counts at specific sites in individual years for various reasons, such as severe weather conditions during the counting period, abandonment of the study site, restricted access, or where counts are repeated in multi-year intervals. The TRIM model imputes missing data using species counts either from surveyed sites with similar environmental characteristics (stratified imputing) or all other sites with available data^37,39^. This process is based on the assumption that changes in populations at non-counted sites are similar to those at counted sites within the same stratum. To derive expected between-year changes in species population sizes, the program fits a log-linear regression model assuming Poisson distribution to time series from counted plots. Finally, we use this model to calculate missing species-specific counts for individual years^37,39^. The resulting time series of species counts with imputed missing values cover the whole period of counts in the national monitoring scheme. These imputed data are then used to estimate annual population sizes from all study plots and to derive population size indices for species^11^.

### European species indices and trends

The individual national indices for a given species are combined to create the European species indices. Subsequently, long-term population size changes (trends) are calculated as the multiplicative linear slopes from species indices and represent an average between-year relative population size change over a predefined period.

The European combination process is very similar to the production of national scheme species indices, but with three differences^40^. Firstly, the indices are calculated using national TRIM output data, consisting of imputed species counts, standard errors per year and covariance matrices. Secondly, species counts are weighted by the most recent species population size estimates (updated every three years) in a given country derived from national bird atlases, official data reports and national experts (http://datazone.birdlife.org/) to account for the country-specific population sizes and thus the unequal contribution of national indices on the European index. Thirdly, missing national time totals due to different start years of the schemes^8^ are imputed using species counts from a set of countries from the same geographical region^6,11^. For this purpose, we divided all national schemes into seven geographic regions – Central & East Europe, East Mediterranean, North Europe, South Europe, Southeast Europe, West Balkan and West Europe^8^. We then use a set of national indices from a given region to impute missing national indices. Therefore, the earliest periods of population size changes are based on data from a reduced number of study plots and schemes.

The species trends are then imputed from species indices for four periods: 1980 onwards, 1990 onwards, 2000 onwards and using only the last ten years of data if the data are available. Despite higher uncertainty of the earliest estimates, we do provide the population index estimates for this period as no alternative and continuous measures of bird population size changes exist for this period.

The uncertainty estimates of indices and trends are presented by the standard error^11,37^ allowing a calculation of 95% confidence limits (±1.96 × standard error). The magnitude of the trend estimates together with their 95% confidence intervals are then used for trend classification into six classes facilitating communication and interpretation of the outputs^37^ (Table 1).

**Table 1:** Classification of the European bird species trends based on the magnitude and uncertainty of the estimates (using 95% confidence intervals).

Class	Description
Steep decline	A trend slope of <0.95 (a decline of more than 5% per year), with the upper confidence limit of the slope <0.95
Moderate decline	A trend slope of ≥0.95 and 1.00 (a decline of no more than 5% per year), with the upper confidence limit of the slope between 0.95 and 1.00.
Stable	A trend slope where the confidence intervals overlap 1 (no significant change), with the lower confidence limit of change >0.95 and upper confidence limit of change <1.05
Uncertain	A trend slope where the confidence intervals overlap 1 (no significant change), with the lower confidence limit of change <0.95 and/or the upper confidence limit of change >1.05
Moderate increase	A trend slope between 1.00 and ≤1.05 (an increase of no more than 5% per year), with the lower confidence limit of the slope between 1.00 and 1.05.
Strong increase	A trend slope of >1.05 (an increase of more than 5% per year), with the lower confidence limit of the slope >1.05

Finally, European species indices and trends are presented only for a group of common and widespread bird species (hereafter ‘common bird species’) meeting two criteria:

1. The estimated breeding population (http://datazone.birdlife.org/) is at least 50 000 pairs in PECBMS Europe (EU countries, Norway, Switzerland and the United Kingdom; Fig. 1). Additionally, Red-billed Chough (*Pyrrhocorax pyrrhocorax*) and Spotted Redshank (*Tringa erythropus*) with population sizes below 50 000 pairs are included, as large parts of their breeding populations are covered in the PECBMS Europe.
2. The estimated breeding population in PECBMS countries providing data for a given species^8^ covers at least 50% of the whole PECBMS Europe breeding population (http://datazone.birdlife.org/).

The resulting datasets of European population size indices and trends consist of relative population changes for 170 common bird species.

### Updates

We aim to maintain the PECBMS database with annual updates. The annual updates will be available through the PECBMS database deposited at the Zenodo repository^8^ to ensure long-term public availability of the data.

## Data Records

The Pan-European Common Bird Monitoring Scheme database is organised into five datasets: (1) European species indices, (2) European species trends, (3) European species trends for three short periods, (4) a list of details on the national monitoring schemes and (5) a matrix of countries providing data for population size estimates of individual species^8^. Individual fields are described in Tables 2–6 and the whole database is freely available at the Zenodo data repository^8^. National-level species indices and uncertainty estimates are also available in the PECBMS database. Due to specific privacy ownership rights, the most recent (2016–2017) Spanish^41^ and Cypriot^42^ data are under Restricted Access and researchers interested in these most recent updates are required to provide a brief description of the data use. The Austrian and Portuguese datasets are publicly available but researchers using these datasets are kindly requested to notify the national scheme coordinators of their use. A list of regularly updated contacts to all national scheme coordinators is provided at the PECBMs website (https://pecbms.info/country/).

**Table 2:** Fields of the European bird population size indices dataset (indices.csv).

Field name	Description
species	scientific name (HBW and BirdLife International 2018)
euring_code	EURING species code (EURING 2018)
year	the year
index	relative population change relative to the reference year
se	standard error of the index value

**Table 3:** Fields of the European bird population size trends dataset (trends.csv).

Field	Description
species	scientific name (HBW and BirdLife International 2018)
euring_code	EURING species code (EURING 2018)
base_year	the reference year
trend	multiplicative slope of the between-year population change
se	standard error of the trend value
class	trend value classification based on its magnitude and uncertainty estimate (Table 1)
note	the note

**Table 4:** Fields of the European bird population size trends dataset for 1990 onwards, 2000 onwards and for the last ten years of the data (trends_short.csv).

Field	Description
species	scientific name (HBW and BirdLife International 2018)
euring_code	EURING species code (EURING 2018)
base_year	the reference year
trend	multiplicative slope of the between-year population change
se	standard error of the trend value
class	trend value classification based on its magnitude and uncertainty estimate (Table 1)

**Table 5:** Fields of the list of the national monitoring schemes (monitoring_schemes.xlsx).

Field	Description
country	country
collaborators	sample size in last year of survey
scheme_name	national monitoring scheme name
count_method	field methods for counting birds
plot_selection	method used for selection of study plots
initial_year	the first year of the data collection
end_year	the last year of the data collection
region	geographic region of the national scheme
reference	reference to the national scheme monitoring program

**Table 6:** Dimensions of the matrix of species and countries providing data to the PECBMS (species_country.csv).

**Rows**	species
**Columns**	country

## Technical Validation

Coordinators of the national monitoring schemes provide all fieldworkers participating in national monitoring schemes with detailed information on counting methods and selection of study plots. Fieldworkers also receive detailed instructions on counting procedures with recommendations to follow guidelines on weather conditions during counts, and time and duration of counts to ensure between-year consistency in bird abundance estimates. Coordinators routinely offer and run practical training sessions and workshops on national fieldwork methods. Moreover, coordinators oversee study plot selection in cases where observers are free to choose their study plots, to prevent oversampling of certain habitat types or regions. Finally, all fieldworkers are advised to carefully check all their records prior to data transfer to the national coordinators. Use of standardized counting protocols, standardized counting conditions^10^ and randomization or supervised study plot selection should yield unbiased year-to-year changes in bird abundances.

The national coordinators carefully check received data for errors caused by data transcription and run TRIM models^37^ combining data records from all study plots and estimating the species’ abundance for the whole scheme. The model outputs are evaluated for credibility using outlier control and a consistency evaluation. Firstly, national coordinators check an outlier report of a national index for each species that indicates very low (<0.5) or very high (>1000) annual index values, very low (1 individual) or very high species counts (>1 × 10^6^ individuals), the number of zero-count sites, the number of sites with missing counts, and the number of sites with more than 10% of the total national species count. The outlier check indicates possible discrepancies in raw data and transcription errors. Secondly, index and trend estimates, species counts, uncertainty estimates and trend classification for a given year are compared to the information from the previous (or any other) year. After evaluating the data credibility, the whole national dataset including indices, trends and uncertainty estimates for each species is transferred to the PECBMS coordination unit. The quality check at the PECBMS level consists of similar steps. Finally, species recorded at very few study plots (i.e. some nocturnal species, rare species, species with a small distribution ranges) are filtered out from the final PECBMS datasets.

The European species’ indices and trends are calculated for the period since 1980; however, the start years differ between schemes (see Methods for details). Therefore, we annually prepare a trend dataset using three additional, shorter periods – 1990 onwards, 2000 onwards and using the last ten years of the data. This yields more robust trend estimates for those periods and allows the comparison of trend estimates between different periods by omitting the earliest years of counts in which only a limited number of schemes were in place and a limited number of fieldworkers recorded data. Despite a higher uncertainty, we present population size change estimates for the earliest periods because there are no alternative data available describing large-scale population size changes in breeding birds for the past four decades.

## Usage Notes

The resulting population indices and trends reflect changes in relative population size, which is our main goal. Our data do not reflect true population estimates because the actual numbers of birds, as assessed at site-level, may be biased in a number of ways. The bias may arise from imperfect detection of birds by fieldworkers, the use of multiple survey protocols in different countries, or the imputation of missing values. But given the fact that within each country the same methods are used at the same sites over time, the data allow us to estimate temporal trends in population sizes.

We advise all PECBMS database users to check notes associated with trend estimates. These notes were added when e.g. a high variation in between-year population sizes was detected, or the trend estimates may have been affected by human activities such as hunting or releasing of captive-bred individuals. For Bluethroat (*Luscinia svecica*), the population indices and trends represent population change of the subspecies *Luscinia svecica svecica* only.

Raw bird counts as well as additional details on the sampling sites can be requested from national scheme coordinators. The updated list of their contact information is available at the PECBMS website (https://pecbms.info/country/). Finally, we encourage anyone planning to use the PECBMS datasets in their study to contact the PECBMS coordination team (https://pecbms.info/) with any questions, especially on the possibilities and limitations of these datasets in relation to the aims of the planned study.

## References

[1] Tilman D (2017). Future threats to biodiversity and pathways to their prevention. Nature.

[2] Costanza R (1997). The value of the world’s ecosystem services and natural capital. Nature.

[3] Ceballos G (2015). Accelerated modern human–induced species losses: Entering the sixth mass extinction. Sci. Adv..

[4] Greenwood JJD (2007). Citizens, science and bird conservation. J. Ornithol..

[5] Barrow, M. A passion for birds: American ornithology after Audubon. (Princeton Press, 1998).

[6] Gregory RD, van Strien A (2010). Wild bird indicators: using composite population trends of birds as measure of environmental health. Ornithol. Sci..

[7] Herrando S (2017). High resolution maps for the second European Breeding Bird Atlas: a first provision of standardised data and pilot modelled maps. Vogelwelt.

[8] Brlík V (2020). Zenodo.

[9] Sauer JR, Link WA, Fallon JE, Pardieck KL, Ziolkowski DJ (2013). The North American Breeding Bird Survey 1966–2011: Summary Analysis and Species Accounts. North Am. Fauna.

[10] Voříšek, P., Klvaňová, A., Wotton, S. & Gregory, R. D. A best practice guide for wild bird monitoring schemes. (JAVA Třeboň, 2008).

[11] Gregory RD (2005). Developing indicators for European birds. Philos. Trans. R. Soc. B.

[12] Gregory RD (2007). Population trends of widespread woodland birds in Europe. Ibis (Lond. 1859)..

[13] Gregory RD (2008). The generation and use of bird population indicators in Europe. Bird Conserv. Int..

[14] Klvaňová A, Voříšek P, Gregory RD, van Strien A, Gmelig Meyling AW (2009). Wild birds as indicators in Europe: latest results from the Pan-European Common Bird Monitoring Scheme(PECBMS). Avocetta.

[15] Gamero A (2017). Tracking Progress Toward EU Biodiversity Strategy Targets: EU Policy Effects in Preserving its Common Farmland Birds. Conserv. Lett..

[16] Butler SJ, Boccaccio L, Gregory RD, Vorisek P, Norris K (2010). Quantifying the impact of land-use change to European farmland bird populations. Agric. Ecosyst. Environ..

[17] Søgaard Jørgensen P (2016). Continent-scale global change attribution in European birds - combining annual and decadal time scales. Glob. Chang. Biol..

[18] Voříšek, P. et al. Trends in abundance and biomass of widespread European farmland birds: how much have we lost? in BOU Proceedings – Lowland Farmland Birds III 24 (2010).

[19] Gregory RD (2009). An Indicator of the Impact of Climatic Change on European Bird Populations. PLoS One.

[20] Pe’er G (2014). EU agricultural reform fails on biodiversity. Science.

[21] Vickery JA (2014). The decline of Afro-Palaearctic migrants and an assessment of potential causes. Ibis.

[22] Inger R (2015). Common European birds are declining rapidly while less abundant species’ numbers are rising. Ecol. Lett..

[23] Stephens PA (2016). Consistent response of bird population changes to climate change on two continents. Science.

[24] Gregory RD, Skorpilova J, Vorisek P, Butler S (2019). An analysis of trends, uncertainty and species selection shows contrasting trends of widespread forest and farmland birds in Europe. Ecol. Indic..

[25] Howard C (2020). Disentangling the relative roles of climate and land cover change in driving the long-term population trends of European migratory birds. Divers. Distrib..

[26] Vorisek P, Gregory RD, Strien AJ (2008). Van & Gmelig Meyling, A. Population trends of 48 common terrestrial bird species in Europe: results from the Pan-European Common Bird Monitoring Scheme. Rev. Catalana d’Ornitologia.

[27] Jiguet F (2010). Population trends of European common birds are predicted by characteristics of their climatic niche. Glob. Chang. Biol..

[28] Butchart S (2010). Global Biodiversity: Indicators of Recent Declines. Science.

[29] Tittensor DP (2014). A mid-term analysis of progress toward international biodiversity targets. Science.

[30] Loh J (2005). The Living Planet Index: using species population time series to track trends in biodiversity. Philos. Trans. R. Soc. B.

[31] McRae L, Deinet S, Freeman R (2017). The Diversity-Weighted Living Planet Index: Controlling for Taxonomic Bias in a Global Biodiversity Indicator. PLoS One.

[32] Eurostat. *Smarter, greener, more inclusive - indicators to support the Europe 2020 strategy*, 10.2785/379691 (2019).

[33] Bibby, C. J., Burgess, N. D., Hill, D. A. & Mustoe, S. Bird Census Techniques. (Academic Press, 2000).

[34] Sutherland, W. Ecological census techniques: A handbook (Cambridge University Press, 1996).

[35] Rosenstock S, Anderson D, Giesen K, Leukering T, Carter M (2002). Landbird counting techniques: current practices and an alternative. Auk.

[36] Van Turnhout CAM (2008). Monitoring common and scarce breeding birds in the Netherlands: applying a post-hoc stratification and weighting procedure to obtain less biased population trends. Rev. Catalana d’Ornitologia.

[37] Pannekoek, J. & van Strien, A. TRIM 3 manual. TRends and Indices for Monitoring data (2001).

[38] Bogaart, P., van der Loo, M. & Pannekoek, J. rtrim: Trends and Indices for Monitoring Data. R package version 2.0.6. https://CRAN.R-project.org/package=rtrim (2018).

[39] Ter Braak, C., van Strien, A., Meijer, R. & Verstrael, T. Bird Numbers 1992. Distribution, Monitoring and Ecological Aspects. in Proceedings 12th International Conference of IBCC and EOAC (1992).

[40] van Strien AJ, Pannekoek J, Gibbons DW (2010). Indexing European bird population trends using results of national monitoring schemes: a trial of a new method. Bird Study.

[41] Brlík V (2021). Zenodo.

[42] Brlík V (2021). Zenodo.

